# Decoding ferroptosis in intracerebral hemorrhage: multidimensional monitoring and integrative perspectives beyond single biomarkers

**DOI:** 10.3389/fneur.2026.1835757

**Published:** 2026-05-18

**Authors:** Ciliang Xiao, Mingchao Deng, Dehong Zhang

**Affiliations:** Department of Clinical Laboratory, Hongya Hospital of Chinese Medicine, Meishan, Sichuan, China

**Keywords:** biomarkers, ferroptosis, intracerebral hemorrhage, multidimensional detection model, prognostic assessment

## Abstract

Although ferroptosis is well-established as a key driver of secondary brain injury following intracerebral hemorrhage (ICH), current monitoring strategies fail to capture its dynamic substrate-execution-amplification network. This failure stems from the susceptibility of single biomarkers to systemic inflammatory confounding and the lack of molecular sensitivity in conventional imaging, ultimately causing a profound disconnect between pathological monitoring and clinical intervention. In light of this, after critically evaluating existing limitations, this review proposes a mechanism-driven, multidimensional integrative monitoring framework: specifically, it aims to eliminate systemic confounding by combining iron metabolism with inflammatory indices (contextualization), track neuronal lipid peroxidation utilizing brain-derived exosomes and F4-neuroprostanes (specificity), and visualize iron deposition by integrating biofluid biomarkers with quantitative susceptibility mapping (spatial resolution). Such multimodal integration represents more than a mere refinement of diagnostic techniques; it is a prerequisite for achieving precision medicine in ICH. Serving as a critical companion diagnostic tool, this framework facilitates the stratification of patients responsive to iron chelation and anti-ferroptotic therapies, thereby propelling clinical management from empirical treatment toward mechanism-directed precision intervention.

## 1 Introduction

Despite the maturation of hematoma evacuation techniques for intracerebral hemorrhage (ICH), a perplexing anatomical-functional mismatch persists in clinical practice: many patients inevitably suffer delayed neurological deterioration even after successful hematoma removal or volume stabilization (1–3). This clinical conundrum compellingly underscores that ICH-induced brain injury is dictated not merely by the mechanical mass effect of the hematoma, but rather by an insidious, continuously evolving secondary biochemical storm (4–6).

Within this pathological cascade, mounting evidence has pinpointed ferroptosis-a form of regulated cell death driven by iron-dependent lipid peroxidation-as the core execution mechanism of secondary brain injury (7–9). The explosive release of hemoglobin and free iron following erythrocyte lysis not only directly catalyzes oxidative stress via the Fenton reaction, but also synergistically inhibits glutathione peroxidase 4 (GPX4) activity and activates the ACSL4 signaling axis, culminating in the irreversible accumulation of lethal lipid peroxides on neuronal membranes (10, 11). Consequently, ferroptosis has emerged as the critical molecular bridge linking hematoma degradation to neuronal loss.

However, despite basic research elucidating the mechanisms of ferroptosis down to the subcellular level, translating these findings into monitoring tools that guide clinical decision-making still faces a formidable translational chasm. The first hurdle is a lack of specificity: although serum ferritin, commonly used in clinical settings, reflects iron burden, as an acute-phase protein, it is highly susceptible to confounding by pulmonary infections or systemic inflammation, failing to accurately map intracerebral pathological alterations (12). Second is detection instability: lipid peroxidation products (e.g., 4-HNE, MDA), which serve as core hallmarks of ferroptosis, are highly prone to autoxidation *in vitro*; the absence of standardized pre-analytical workflows leads to poor reproducibility of assay results (13, 14). Finally, there are spatiotemporal limitations: single peripheral blood metrics struggle to reflect the localized pathological state within the blood–brain barrier (BBB), creating an informational disconnect between peripheral assays and central pathology (15).

It is precisely due to these limitations that efforts to identify a single gold-standard biomarker have largely ended in failure. To overcome this obstacle, this review moves beyond the mere enumeration of individual metrics to propose a mechanism-based, multidimensional integrative monitoring strategy. We advocate for the establishment of a multimodal framework encompassing the substrate (iron metabolism), execution (lipid peroxidation), and amplification (inflammatory crosstalk) phases. This involves enhancing the specificity of ferritin through the joint calibration of the inflammatory background, achieving precise tracing of brain-derived injury via exosome technology, and realizing the visualization of pathology in combination with quantitative imaging. This paradigm shift aims to provide a core translational tool for identifying ferroptosis-dominant patients, guiding the precise administration of iron chelators, and evaluating the efficacy of novel neuroprotectants, thereby propelling the clinical management of ICH from empirical treatment to a mechanism-directed precision medicine paradigm.

## 2 From molecular cascades to diagnostic targets: a substrate-execution-amplification pathological network

Secondary brain injury following ICH is not a stochastic process; rather, it adheres to a highly orchestrated spatiotemporal progression of ferroptosis, characterized by substrate accumulation, program execution, and signal amplification (16). Deconstructing this network serves as the theoretical prerequisite for identifying highly specific biomarkers.

### 2.1 The substrate dimension: iron metabolism

Erythrocyte lysis acts as the primary initiator of this pathological cascade (4, 17–19). The liberated heme initially functions as a molecular trigger, activating downstream transcriptional programs; subsequently, the explosive release of free iron overwhelms the buffering capacity of local ferritin (16, 20). Crucially, this process is not confined to the local microenvironment. Through the brain-liver axis, it provokes a profound surge in systemic hepcidin, driving a complete reprogramming of systemic iron metabolism (21, 22). This systemic reverberation establishes the pathological rationale for utilizing hepcidin to monitor systemic iron sequestration.

### 2.2 The execution dimension: lipid peroxidation

The crux of ferroptosis lies within a fulminant lipid peroxidation storm (23–26). Driven by the concomitant inhibition of the master antioxidant axis and the activation of ACSL4, lethal lipid peroxides inexorably accumulate on neuronal membranes (20). Under physiological conditions, the cystine/glutamate antiporter (whose core subunit is SLC7A11) imports cystine to synthesize glutathione (GSH), serving as the essential fuel for GPX4 to neutralize lipid peroxides. Following ICH, the profound suppression of this SLC7A11/GSH/GPX4 defense network accelerates the execution of ferroptosis. Distinct from non-specific necrosis, this peroxidative process exhibits high substrate specificity, predominantly targeting polyunsaturated fatty acids (PUFAs). Furthermore, the resultant byproducts (e.g., 4-HNE) can either passively leak across the compromised BBB or be actively secreted via exosomes (27–31). These distinct dissemination routes validate the feasibility of employing highly specific lipid markers and exosomal profiling for precise cellular tracing.

### 2.3 The amplification dimension: neuroinflammation

**Figure 1:**
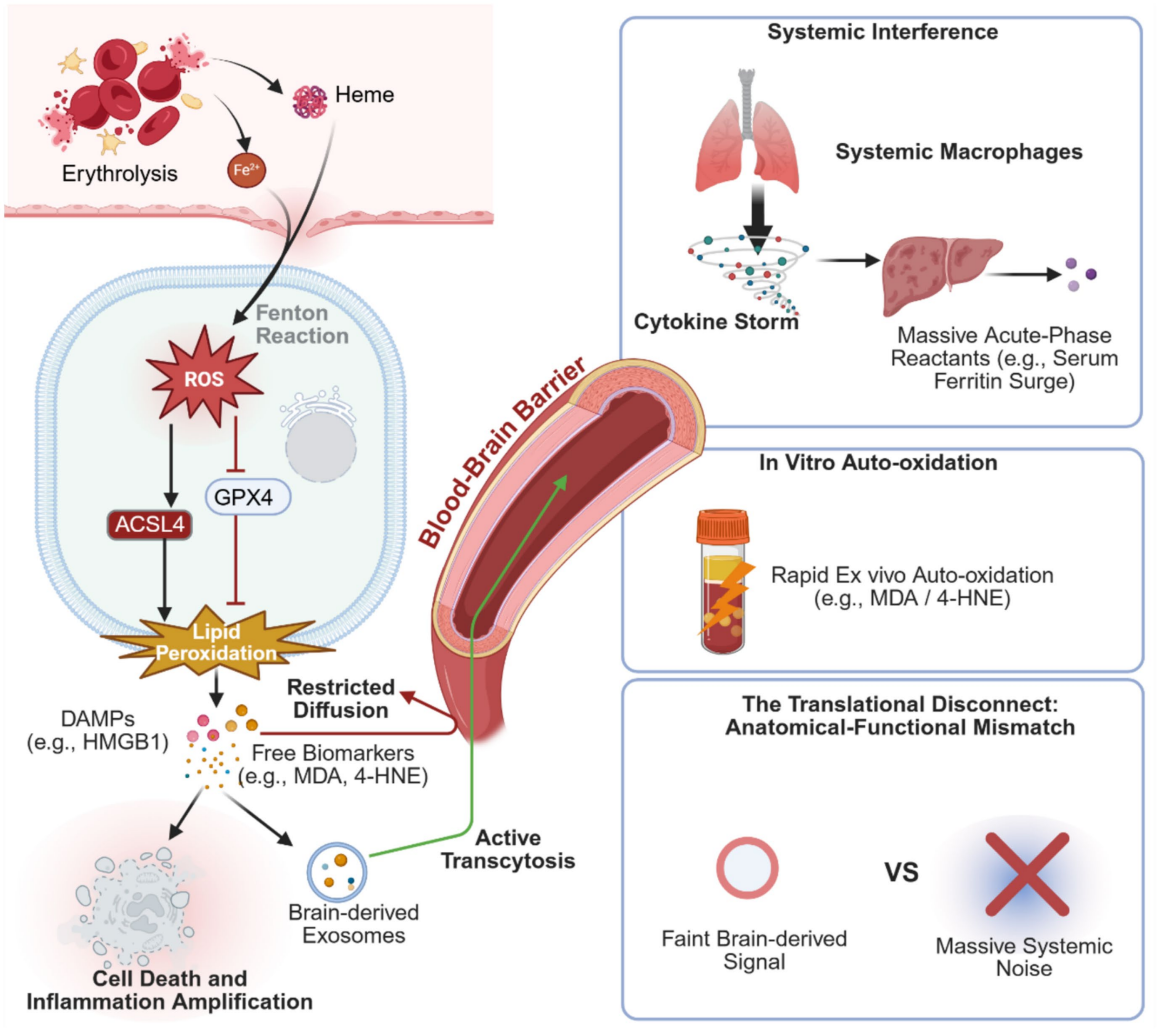
Pathological mechanisms of ferroptosis following ICH and the monitoring limitations of single peripheral blood biomarkers. The ferroptotic cascade: Following ICH, erythrocyte lysis releases free iron and heme, inducing local oxidative stress via the Fenton reaction. This process inhibits GPX4 activity and activates the ACSL4 pathway, culminating in the lipid peroxidation of PUFAs on neuronal membranes. Subsequent cellular damage triggers the release of DAMPs, provoking secondary neuroinflammation. Limitations of conventional monitoring: Clinical monitoring reliant on single peripheral blood biomarkers (e.g., MDA, 4-HNE, or serum ferritin) faces three primary constraints. First, the BBB severely impedes the passive diffusion of free central biochemical signals (e.g., free lipid peroxidation products) into the circulation, resulting in significantly attenuated peripheral concentrations. This physiological blockade precisely highlights the necessity of tracking active vesicular carriers, such as exosomes, which are capable of transcytosis. Second, conventional lipid peroxidation products are highly prone to *in vitro* autoxidation during sample processing, compromising assay reproducibility. Finally, acute-phase reactants such as serum ferritin are highly susceptible to non-specific confounding by acute systemic inflammation and common complications (e.g., pulmonary infections), rendering peripheral readouts inadequate for accurately quantifying localized intracerebral pathology.

Ferroptosis and the innate immune response engage in a malignant and reciprocal crosstalk (32–35). During the microglial metabolism of heme, the hyperactivation of heme oxygenase-1-initially a compensatory detoxification mechanism-paradoxically exacerbates intracellular iron overload and fuels an inflammatory storm (11). Furthermore, damage-associated molecular patterns (DAMPs, such as HMGB1) released from dying cells relentlessly promote neutrophil infiltration, further exacerbating BBB disruption (27). This inextricable co-occurrence of iron dyshomeostasis and inflammation firmly dictates the absolute necessity of integrating inflammatory indices (e.g., NLR) for background calibration, as proposed in our primary monitoring strategy (Figure 1) (36).

## 3 Strategy I: Distinguishing signal from noise-correcting systemic noise via multiparametric coupling

In constructing a prognostic evaluation framework for ICH, the foremost challenge lies in decoupling the confounding effects of systemic inflammation from iron metabolism indices. Although baseline levels of serum ferritin-a classical biomarker of iron burden-have been independently correlated with perihematomal edema expansion and poor clinical outcomes, its inherent nature as an acute-phase reactant renders it highly susceptible to non-specific elevation driven by common post-ICH complications (e.g., pulmonary or urinary tract infections) (37, 38). This diminished signal-to-noise ratio means that isolated ferritin measurements cannot reliably distinguish between a generalized inflammatory storm and genuine pathological iron overload. To circumvent this limitation, we propose an integrative calibration strategy that couples inflammatory and iron metabolism indices. First, we advocate integrating the neutrophil-to-lymphocyte ratio (NLR) as a background calibration factor. The NLR not only reflects the early systemic inflammatory burden and the risk of in-hospital mortality, but also serves as a crucial reference frame to determine whether elevated ferritin stems from concurrent infections (39). Indeed, prospective studies have demonstrated that profiling a multidimensional metabolic panel-combining iron, ferritin, and transferrin-yields significantly higher predictive accuracy for long-term outcomes, such as cognitive impairment, compared to any single metric (40). Second, we introduce hepcidin as a mechanism-specific indicator. As the master negative feedback regulator of systemic iron homeostasis, the pathological surge of hepcidin following ICH induces the degradation of ferroportin, thereby precipitating a systemic iron sequestration effect (21, 22). Clinical evidence reveals a strong positive correlation between serum hepcidin levels and intracerebral iron deposition. However, it is crucial to note that hepcidin, much like ferritin, is not exclusively specific to ICH. Its expression can be significantly confounded by widespread systemic conditions such as anemia and inflammation (41). This physiological reality underscores why seminal studies demonstrating hepcidin’s prognostic value in ICH required strict exclusion criteria to eliminate these systemic confounders (21). Therefore, isolated hepcidin measurements in unselected populations are insufficient. Its true diagnostic value for central metabolic reprogramming can only be realized within our proposed multidimensional framework-coupling rigorous clinical screening (e.g., excluding anemia) with inflammatory indices (e.g., NLR) to calibrate for systemic noise. Therefore, establishing a “Ferritin-NLR-Hepcidin” tripartite evaluation model represents the critical first step in transitioning from empirical observation to mechanism-specific monitoring.

## 4 Strategy II: Precise tracing-targeting central signals via specific lipid biomarkers and exosomes

**Figure 2:**
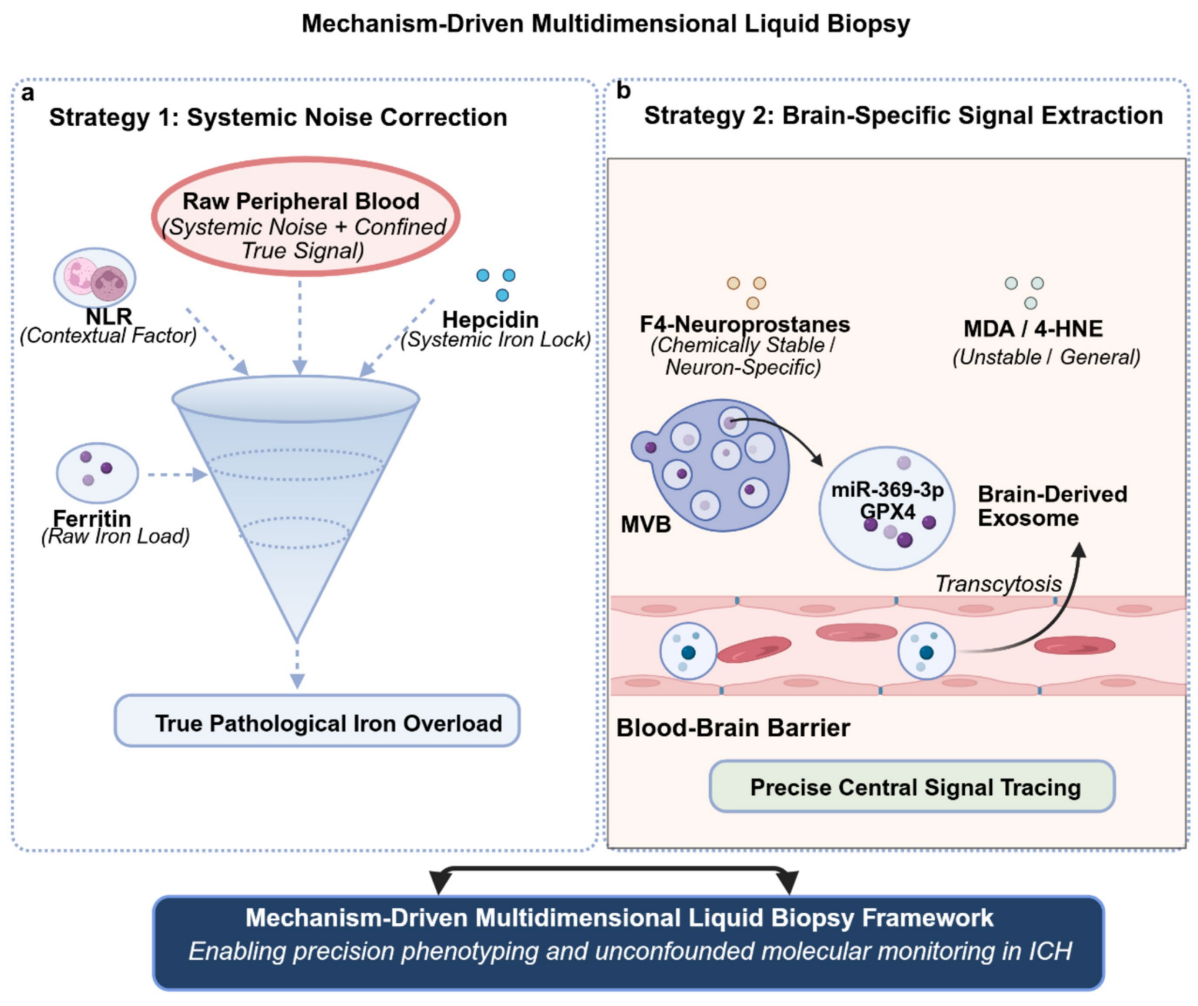
Multidimensional liquid biopsy monitoring model for ferroptosis following intracerebral hemorrhage. **(a)** Iron metabolism and systemic inflammatory calibration strategy: Construction of a ferritin-NLR-hepcidin multiparametric evaluation model. By employing the NLR as a calibration factor for systemic inflammatory burden, in conjunction with hepcidin—the master negative feedback regulator of systemic iron homeostasis—this approach circumvents the confounding effects of non-specific infections on ferritin expression, thereby enabling a specific assessment of the patient’s pathological iron overload. **(b)** Central signal extraction: Substituting conventional pan-oxidation metrics with F_4_-neuroprostanes, a neuron-specific lipid peroxidation product exhibiting superior chemical stability. Concurrently, profiling brain-derived exosomes that actively traverse the BBB, alongside their encapsulated molecular cargo (e.g., GPX4 protein or specific miRNAs), facilitates the non-invasive and highly specific evaluation of central neuronal lipid peroxidation and the integrity of the antioxidant defense system.

The signal dilution effect across the BBB represents another formidable translational chasm for peripheral blood biomarkers. Theoretically, cerebrospinal fluid (CSF) analysis offers a direct, unconfounded window into central iron metabolism and lipid peroxidation, elegantly bypassing both the BBB and systemic inflammation (42). Recent evidence robustly demonstrates that CSF iron and Fe^2+^ concentrations are strongly correlated with adverse outcomes, such as shunt dependency and hydrocephalus, particularly in ICH patients with intraventricular extension (43, 44). However, a critical translational barrier remains: routine CSF sampling via lumbar puncture is frequently contraindicated in acute ICH due to elevated intracranial pressure and the severe risk of cerebral herniation. Moreover, external ventricular drains are strictly reserved for a subset of patients with severe intraventricular hemorrhage. The highly invasive nature and restricted clinical applicability of CSF collection preclude its use as a universal, daily monitoring tool. This stark clinical reality further amplifies the urgent necessity of developing advanced, non-invasive peripheral blood strategies-such as capturing brain-derived exosomes and specific lipid biomarkers-that can accurately mirror central pathology without the risks associated with CSF sampling. Although traditional lipid peroxidation products such as malondialdehyde (MDA) positively correlate with National Institutes of Health Stroke Scale scores, they conspicuously lack tissue specificity. Confounded by systemic oxidative stress factors-including diabetes and dietary variations-they fail to accurately map intracerebral pathology (45, 46). Furthermore, *in vitro* autoxidation during sample processing frequently induces artefactual elevations in unstable metrics, such as F2-isoprostanes (47). Addressing this critical bottleneck, this review advocates a paradigm shift from universal metrics to brain-specific biomarkers. First, we focus on F4-neuroprostanes. As specific oxidation products of docosahexaenoic acid-which is highly enriched in neuronal membranes-they exhibit superior chemical stability compared to MDA and enable a more precise quantification of neuronal membrane disintegration (48). To accurately quantify these trace lipid metabolites, LC–MS/MS currently serves as the gold-standard detection method. In terms of pre-analytical feasibility, we recommend utilizing plasma or serum samples strictly cryopreserved at −80 °C-ideally supplemented with antioxidants immediately upon collection-to absolutely preclude any ex vivo autoxidation. Importantly, however, we must explicitly acknowledge that while the theoretical rationale for F4-neuroprostanes is robust, direct clinical validation data specifically derived from acute ICH patient cohorts are currently lacking. Bridging this critical evidence gap through prospective observational studies to verify its precise temporal dynamics in human ICH constitutes a paramount focus for future translational research. Second, we propose utilizing exosomes as dynamic nanovesicles that bidirectionally traverse the BBB, facilitating both non-invasive liquid biopsies and targeted interventions (28). For diagnostic monitoring (brain-to-blood efflux), recent advanced profiling reveals that distinct cell types within the injured central nervous system secrete exosomes carrying specific molecular signatures into the systemic circulation. For instance, single-cell sequencing has identified that foam cells release exosomes enriched with miRNA Novel-3 during ischemic stroke (29). Similarly, specific endogenous exosomal cargoes such as miR-369-3p have been shown to mirror the pathological modulation of the microglial iNOS/GPX4 axis (31). Conversely, exploiting the influx transport across the BBB, engineered exosomes-such as those derived from mesenchymal stem cells-can be administered systemically to deliver therapeutic cargoes (e.g., miR-1228-5p) into the brain for neuroprotection (30). These nanovesicles not only comprehensively encapsulate upstream regulatory information (such as the status of the iNOS/GPX4 axis) but also shield their cargo from enzymatic degradation via their robust lipid bilayer (31). However, capturing these central signals in the periphery faces a formidable methodological hurdle: true brain-derived exosomes constitute only a minuscule fraction of the total circulating pool. To overcome this, future translational efforts must employ targeted immunoaffinity enrichment utilizing specific surface markers-such as L1 Cell Adhesion Molecule (L1CAM) for neuronal origins or GLAST for astrocytes-to rigorously verify their brain origin prior to downstream cargo analysis. Furthermore, we must explicitly acknowledge that most current evidence regarding these specific exosomal signatures (e.g., miR-369-3p) derives from ischemic stroke or subarachnoid hemorrhage models. Whether the exosomal cargo profiles in ICH perfectly mirror these models remains to be directly validated. Therefore, the application of brain-specific exosomal profiling in ICH currently serves as a compelling, theoretically sound hypothesis rather than an established clinical reality, representing a pivotal trajectory for future empirical investigations. Consequently, profiling exosome-derived GPX4 or specific miRNAs enables a non-invasive, precise, and real-time interrogation of the collapsing intracerebral antioxidant defense network (Figure 2) (49).

## 5 Strategy III: Macro–micro corroboration-cross-dimensional integration of liquid biopsies and quantitative imaging

**Figure 3:**
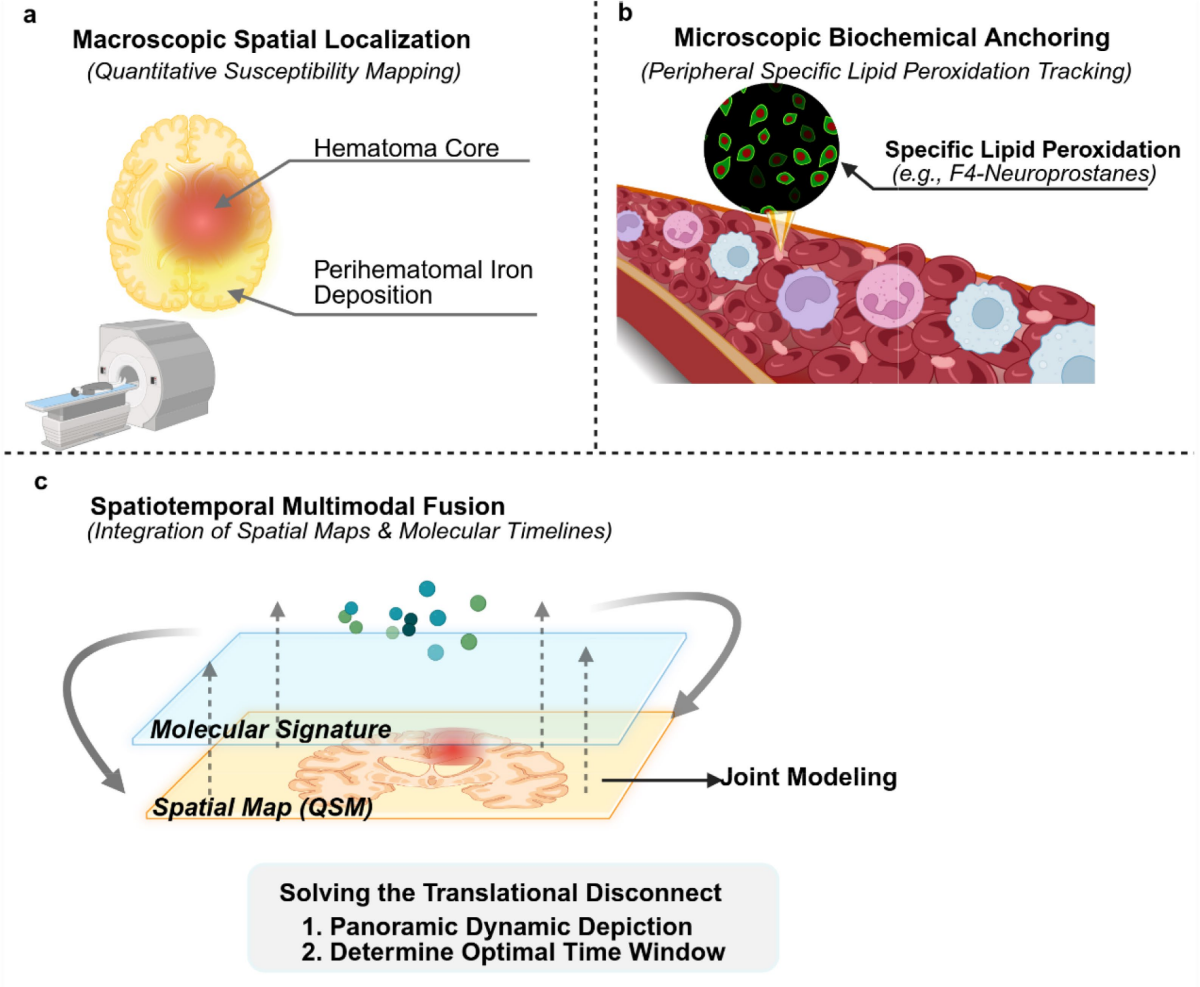
A multimodal integration framework combining biofluid biomarkers with QSM. **(a)** Quantification of spatial distribution: QSM enables the non-invasive, high-spatial-resolution, three-dimensional quantitative mapping of free iron deposition within the perihematomal brain tissue, pinpointing the precise anatomical localization and concentration of iron dyshomeostasis. **(b)** Molecular-level characterization: Peripheral blood-based profiling of exosomes and specific lipid biomarkers (e.g., F4-neuroprostanes) furnishes molecular metabolic readouts that correspond with neuroimaging findings, reflecting the magnitude of ferroptosis at a microscopic level. **(c)** Cross-dimensional data fusion: Integrating spatial structural parameters derived from QSM with biochemical biomarker concentrations obtained via liquid biopsies facilitates the construction of a multimodal evaluation model. This integrative paradigm successfully compensates for the low molecular sensitivity of conventional neuroimaging and the spatial agnosia of hematological assays, providing an objective, visualized rationale for assessing hematoma resolution and the severity of secondary brain injury.

Unidimensional assays inherently suffer from diagnostic blind spots: blood-based metrics provide systemic biochemical readouts but critically lack spatial resolution, whereas conventional CT/MRI, despite localizing the macroscopic hematoma, remain oblivious to microscopic molecular events. To resolve the prevalent clinico-radiological paradox, it is imperative to construct a multimodal integration strategy that bridges liquid biopsies with neuroimaging (3). The crux of this strategy lies in leveraging quantitative susceptibility mapping (QSM) as an indispensable bridge connecting macroscopic lesions to microscopic metabolic alterations. QSM enables the non-invasive quantification of both the concentration and spatial distribution of iron deposition within the perihematomal brain tissue (50). Study has corroborated that integrating MRI-captured signatures of erythrocyte lysis with serum iron metabolism parameters significantly enhances the predictive efficacy for white matter injury risk (2). Indeed, previous studies have successfully utilized CSF lipid peroxides (e.g., F2-isoprostanes) as molecular probes in joint modeling with neuroimaging to precisely identify delayed ischemic events (42). Building upon this robust conceptual foundation, a more forward-looking, non-invasive approach for ICH would involve translating this joint-modeling paradigm to the periphery. Specifically, this entails utilizing stable peripheral blood markers, such as F4-neuroprostanes, as circulating molecular probes to be integrated with QSM features. Ultimately, this macro–micro corroboration paradigm not only achieves a dynamic, panoramic delineation of hematoma resolution, iron release, and cerebral edema, but also provides a visualized, objective rationale for determining the optimal therapeutic window for iron chelation interventions (Figure 3).

## 6 Clinical translation: transitioning from empirical therapy to companion diagnostics

The ultimate objective of establishing a multimodal monitoring strategy extends far beyond mere prognostic scoring; it is fundamentally designed to guide individualized clinical interventions. The failure of previous neuroprotective therapies for ICH-particularly clinical trials of iron chelators such as deferoxamine (e.g., the i-DEF trial)-to translate into definitive evidence of improved patient outcomes can be largely attributed to the absence of biomarker-driven patient stratification strategies (51). The profound pathophysiological heterogeneity of iron toxicity dictates that a precise assessment of the metabolic state must precede treatment, definitively precluding the indiscriminate administration of drugs (51).

### 6.1 Companion diagnostics: precision enrollment for iron chelation and anti-ferroptotic therapies

A one-size-fits-all therapeutic paradigm egregiously overlooks the vast inter-individual variability in iron metabolism. The multidimensional evaluation framework proposed in this review can serve as a critical companion diagnostic tool to identify true responder subgroups. Clinicians should primarily focus on patients exhibiting a phenotype of high iron burden coupled with depleted antioxidant reserves-specifically, those demonstrating pronounced perihematomal iron deposition on neuroimaging, alongside hematological indices revealing an aberrant surge in hepcidin and diminished exosomal GPX4 levels. For this specific ferroptosis-dominant phenotype, iron chelation therapy would theoretically yield maximal therapeutic benefit. Conversely, for patients with inflammation-dominant profiles and low iron burden, the indiscriminate use of chelators may induce adverse effects by interfering with normal physiological iron metabolism. This mechanism-based screening strategy represents a pivotal step toward enhancing the success rate of future translational clinical trials. Furthermore, the expression level of mitochondrial ferritin has been shown to inhibit neuronal ferroptosis via the NDRG1/YAP pathway, suggesting that monitoring peripheral blood markers of mitochondrial function could also serve as an ancillary criterion for initiating targeted therapy (52).

### 6.2 Efficacy monitoring: mechanism-based dynamic feedback and pharmacological evaluation

**Figure 4:**
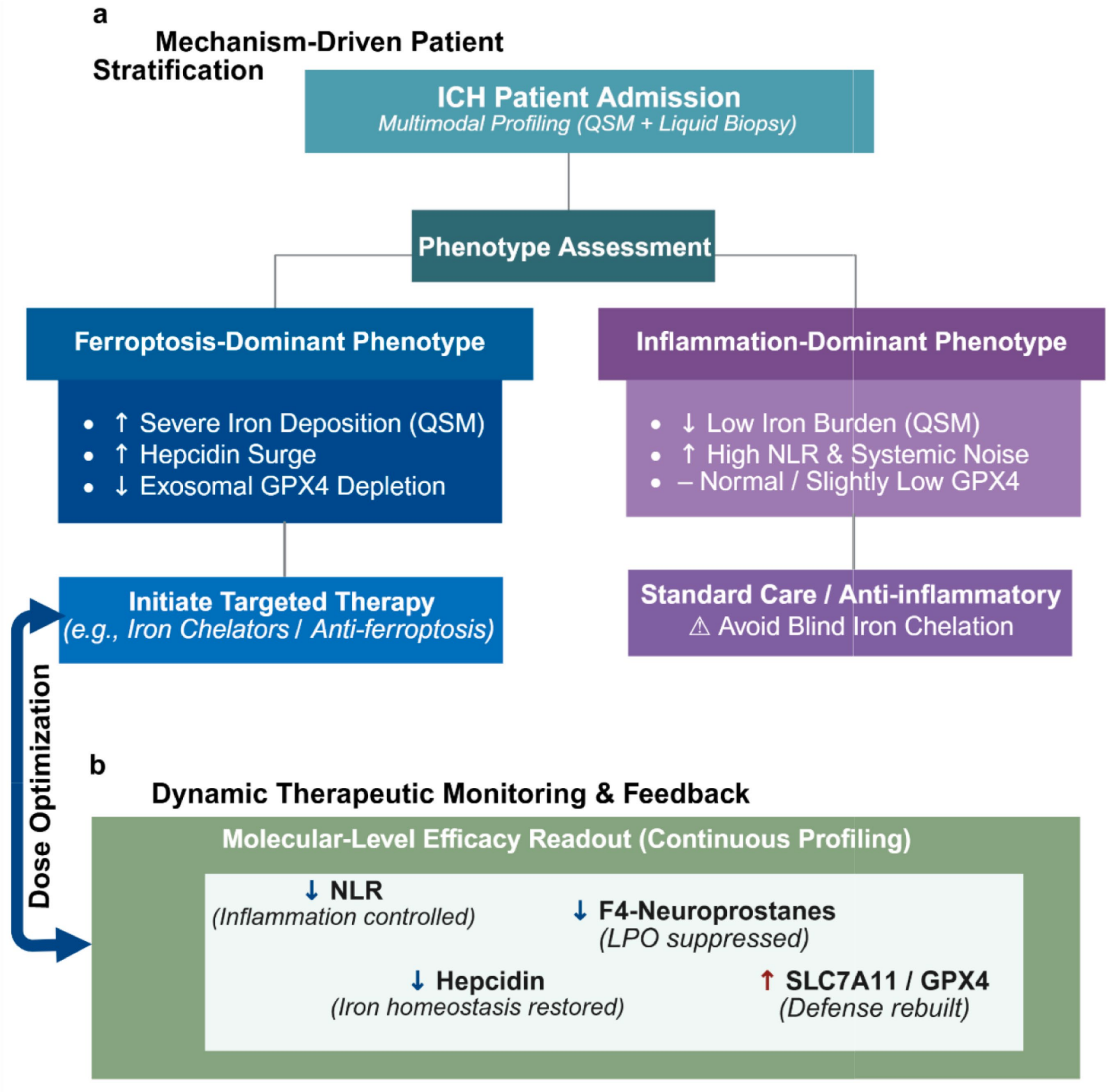
Clinical application pathway of the multidimensional ferroptosis monitoring model in companion diagnostics and targeted therapy for ICH. **(a)** Patient stratification based on biochemical phenotypes: The multimodal model is utilized to evaluate the pathological phenotypes of ICH patients upon admission. Patients demonstrating pronounced perihematomal iron deposition on QSM, coupled with an aberrant surge in peripheral hepcidin and diminished exosomal GPX4 levels, are delineated as the “ferroptosis-dominant” subgroup. This cohort represents the primary target population for iron chelators or anti-ferroptotic therapies (e.g., deferoxamine). Conversely, for patients lacking this combined signature, iron chelation must be approached with stringent caution to mitigate the risk of disrupting physiological iron metabolism. **(b)** Dynamic monitoring of therapeutic efficacy: during the administration of novel neuroprotectants (e.g., edaravone dexborneol) or multi-target combination therapies (e.g., cinnamaldehyde plus deferoxamine), serial assessments are performed for inflammatory indices (NLR reduction), iron metabolism parameters (hepcidin normalization), and specific lipid peroxidation products (a decline in F4-neuroprostanes concurrent with a rebound in SLC7A11 expression). The synchronous transformation of this multidimensional biomarker panel provides direct biological evidence of pharmacological target engagement, thereby guiding clinicians in individualized dose optimization and therapeutic evaluation.

Beyond screening suitable patients, this multimodal model provides molecular readouts for evaluating the efficacy of novel neuroprotectants, thereby overcoming the inherent latency associated with relying solely on clinical scales. Taking the novel free radical scavenger edaravone dexborneol as an example, previous studies have rigorously demonstrated its neuroprotective benefits by suppressing 4-HNE-associated oxidative stress and apoptosis in subarachnoid hemorrhage (13), as well as inhibiting NLRP3 inflammasome-induced pyroptosis in ischemic stroke (53). Given its potent capacity to attenuate lipid peroxidation across diverse stroke models, applying our proposed multidimensional framework to investigate whether this agent concurrently modulates the ferroptotic cascade in ICH represents a compelling direction for future pharmacological evaluation. During the course of treatment, if a decline in specific lipid peroxidation markers (e.g., F4-neuroprostanes) within the model is observed to synchronize with a rebound in antioxidant indices (e.g., SLC7A11), this provides direct evidence of pharmacological efficacy at the molecular level. Regarding multi-target combination therapies, such as the co-administration of cinnamaldehyde and deferoxamine, they exhibit a stronger synergistic anti-inflammatory and anti-ferroptotic effect than monotherapy (12). These complex pharmacological interactions can be quantitatively evaluated by monitoring the synchronous improvement of the inflammatory axis (e.g., a decrease in NLR) and the iron metabolism axis (e.g., the normalization of hepcidin) within the model (12). By longitudinally tracking the temporal trajectories of these biomarkers within the therapeutic window, clinicians can promptly adjust drug dosages or combination strategies, facilitating a definitive transition from empirical dosing to biomarker-guided precision therapy (Figure 4).

### 6.3 Prospective validation concepts and algorithmic integration

Although the theoretical framework of this multidimensional model is robust, transitioning it into a reliable clinical assay necessitates explicit data fusion algorithms, standardized detection protocols, and rigorous prospective validation. To achieve this, cross-dimensional data should not be assessed in simple parallel; instead, variables like the NLR and ferritin must be integrated via interaction terms within a multivariable logistic regression model to construct a composite ferroptosis risk nomogram, while machine learning algorithms (e.g., Random Forest or Support Vector Machines) should be employed to fuse high-dimensional spatial QSM parameters with longitudinal blood biomarker trajectories. Furthermore, ensuring cross-center reproducibility demands strict adherence to MISEV guidelines for exosomal assays, specifically utilizing size-exclusion chromatography for isolation, coupled with ddPCR and ultra-sensitive ELISA for precise molecular quantification. Ultimately, validating this comprehensive algorithm requires a multicenter, prospective cohort design, typically necessitating a discovery cohort of 250 to 300 patients meticulously stratified by baseline hematoma characteristics, paired with an indispensable independent external validation cohort of 100 to 150 patients to definitively confirm the model’s generalizability, calibration, and clinical translational value.

## 7 Perspectives

Although the widespread clinical translation of multimodal monitoring strategies still awaits further technological breakthroughs-such as the application of single-cell multi-omics to decode cell-specific exosomes (e.g., Novel-3) and the development of microfluidic point-of-care testing devices for ultra-sensitive, rapid screening-the integrative framework proposed in this review fundamentally circumvents the systemic confounding and spatiotemporal limitations inherent to single-biomarker assays. Grounded in the principles of distinguishing signal from noise (via integrated inflammatory calibration), precise tracing (via exosomes and specific lipid biomarkers), and macro–micro corroboration (the fusion of liquid biopsies with QSM imaging), this strategy offers a robust solution to the prevalent clinico-radiological paradox. Furthermore, it firmly establishes its critical value as a companion diagnostic tool for iron chelators and targeted nanotherapeutics, ultimately propelling the clinical management of ICH from empirical supportive care toward a mechanism-directed precision medicine paradigm.
